# Genome-wide profiling of alternative splicing in glioblastoma and their clinical value

**DOI:** 10.1186/s12885-021-08681-z

**Published:** 2021-08-26

**Authors:** Youwei Li, Dongsheng Guo

**Affiliations:** Department of Neurosurgery, Tongji Hospital, Tongji Medical College, Huazhong University of Science and Technology, Wuhan, Hubei People’s Republic of China

**Keywords:** Glioblastoma, Alternative splicing, RNA-Seq, Prognosis

## Abstract

**Background:**

Alternative splicing (AS), one of the main post-transcriptional biological regulation mechanisms, plays a key role in the progression of glioblastoma (GBM). Systematic AS profiling in GBM is limited and urgently needed.

**Methods:**

TCGA SpliceSeq data and the corresponding clinical data were downloaded from the TCGA data portal. Survival-related AS events were identified through Kaplan–Meier survival analysis and univariate Cox analysis. Then, splicing correlation network was constructed based on these AS events and associated splicing factors. LASSO regression followed by multivariate Cox analysis was performed to validate independent AS biomarkers and to construct a risk prediction model. Enrichment analysis was subsequently conducted to explore potential signaling pathways of these AS events.

**Results:**

A total of 132 TCGA GBM samples and 45,610 AS events were included in our study, among which 416 survival-related AS events were identified. An AS correlation network, including 54 AS events and 94 splicing factors, was constructed, and further functional enrichment was performed. Moreover, the novel risk prediction model we constructed displayed moderate performance (the area under the curves were > 0.7) at both one, two and three years.

**Conclusions:**

Survival-related AS events may be vital factors of both biological function and prognosis. Our findings in this study can deepen the understanding of the complicated mechanisms of AS in GBM and provide novel insights for further study. Moreover, our risk prediction model is ready for preliminary clinical applications. Further verification is required.

**Supplementary Information:**

The online version contains supplementary material available at 10.1186/s12885-021-08681-z.

## Background

Glioblastoma (GBM) is the most common intrinsic malignant tumor of the nervous system and the most malignant glioma [1, 2]. Traditionally, the treatment of GBM mainly includes surgical resection and postoperative involved-field adjuvant radiotherapy and chemotherapy [3, 4]. Some types of anti-tumor compounds that target specific molecules or pathways are also being used in existing treatments. Unfortunately, large-scale studies have failed to demonstrate that these potential therapeutic targets can alter the course of the disease or improve patient outcomes because of various known and unknown mechanisms [5–10]. As a result, the clinical outcome of GBM is unsatisfactory, with a five-year survival rate of less than 5% and an average survival time of approximately 15 months after diagnosis [11]. More seriously, its characteristics of high invasive ability and rapid invasive growth make it difficult to perform total surgical resection [12]. Recent studies of GBM have shown that GBM is a highly heterogeneous tumor with complicated genetic alterations, and its characteristics, such as invasiveness, cell apoptosis, angiogenesis promotion and tumor drug resistance, constitute a complex process that is related to alterations of many genes [13]. Thus, it is important to further understand the biological mechanisms of GBM regulation and the relationship between GBM and its clinical characteristics, which will be conducive for improving the treatment strategy and outcomes of patients.

Alternative splicing (AS) is a common phenomenon in eukaryotes and occurs in approximately 90% of human genes [14]. Currently, around 20,000 protein-coding genes have been found in the human genome, but the number of mature mRNAs (message RNAs) in transcriptomics vastly exceeds the number of protein-coding genes (the current version of GENCODE (GENCODE 31) identified 82,141 different mature mRNA sequences) [15, 16]. AS, selectively removing special sequences of precursor RNA to produce different mature mRNA isoforms, is one of the main mechanisms of RNA polymorphism. As a vital part of the post-transcriptional biological regulation mechanism, AS plays a key role in promoting protein polymorphism by altering functional domains and modification of proteins [17, 18]. For the same coding gene, its corresponding protein isoforms can perform different or even completely opposite functions, thus playing a vital role in regulating complex biological functions [19]. In GBM, changes in the balance of splicing isoforms or the production of new splicing isoforms can alter the expression of the corresponding proteins and promote the generation of various malignant phenotypes. For example, C-CBL is an E3 ubiquitin protein ligase involved in cell signal transduction. Splicing isoforms caused by exon skipping of C-CBL can lead to tumor growth whereas C-CBL itself can serve as an inhibitor of cell proliferation in normal tissues [20]. Similarly, the upregulation of MYO1B-fl caused by splice-switching promotes cell proliferation and changes of the cytoskeleton, thus promoting the growth of GBM [21]. Therefore, cancer-specific splicing variants may be used as diagnostic, prognostic and predictive biomarkers as well as therapeutic targets.

The rapid development of high-throughput sequencing technology has allowed us to focus on the links between various molecules and pathways in diseases. Moreover, it also provides us a new perspective to systematically understand the complex molecular mechanism of GBM as well as to search for potential therapeutic targets and prognostic markers. For example, a study based on the whole genome and corresponding clinical data of The Cancer Genome Atlas (TCGA) database indicated that copy number variation (CNV) can be used as a potential clinical prognostic factor [22]. Complementally, research involved long non-coding RNA (lncRNA) expression and DNA methylation have been widely conducted in GBM [23, 24]. These studies based on high-throughput sequencing techniques identified pathways involved in GBM and potential therapeutic targets as well as prognostic factors. Moreover, these results suggest that high-throughput sequencing is appropriate and effective for understanding GBM, which is a highly heterogeneous tumor. Considering the universality of AS in GBM and its complex biological mechanism, genome-wide AS analysis can deepen our understanding of the mechanism of the oncogenesis and progression of AS in GBM. However, unlike other genomic data available at the levels of gene expression, copy number variation and DNA methylation, research focusing on AS is limited and urgently needed.

In this study, systematic analysis was performed to understand the correlation between genome-wide AS data and clinical outcomes. Based on the corresponding SpliceSeq data from the TCGA database, we identified patient outcome associated AS events and constructed an AS associated network and an AS prognosis model. We also analyzed the potential pathway through Gene set enrichment analysis (GSEA) and its predictive value. These findings revealed new potential therapeutic targets and prognostic factor and provided a new perspective for understanding the molecular mechanism of AS and its clinical application in GBM.

## Methods

### Data curation process

**Fig. 1:**
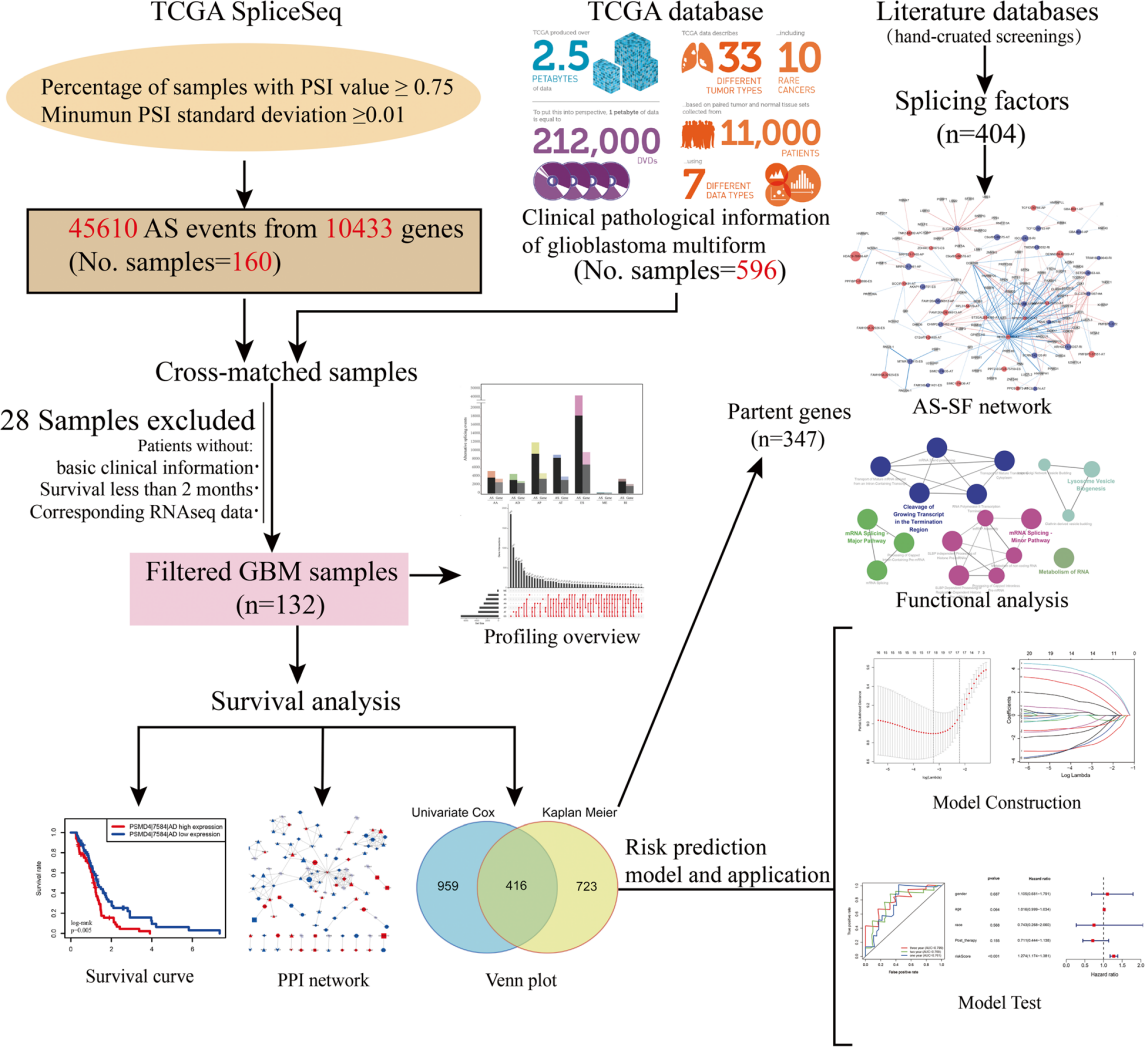
Flowchart for systematically profiling the alternative splicing of GBM in a large-scale RNA-Seq data

Transcriptional sequence data and corresponding clinical data of GBM cases were downloaded from the TCGA data portal [25]. SpliceSeq, a tool that can be used to evaluate the mRNA splicing patterns, was used to analyze our TCGA RNASeq data as previous described [26]. The Percent Spliced In (PSI) value is an intuitive ratio for quantifying splicing events. PSI is the ratio of normalized read counts indicating inclusion of a transcript element over the total normalized reads for that event (both inclusion and exclusion reads), which has a value between 0 and 1. Using the identification number of TCGA, SpliceSeq resources and clinical data were cross-referenced. All cases with TCGA data that meet the following criteria were included: 1. An available histological diagnosis of GBM; 2. Patients with available SpliceSeq data; 3. Patients with basic clinical information including survival status and survival time; and 4. Patients who survived for more than two months after the initial diagnosis. To obtain reliable data, we strictly filtered the downloaded PSI values of all samples (percentage of samples with PSI value ≥0.75, minimum PSI standard deviation ≥0.01). All AS events are classified into seven types, including alternate acceptor site (AA), alternate donor (AD), alternate promoter (AP), alternate terminator (AT), exon skip (ES), mutually exclusive exons (ME), and retained intron (RI). An UpSet plot was used to show the seven different patterns of AS events in all gene concentrations [27]. Details of the research design are shown in Fig. 1 as a flowchart.

### Identification of prognostic AS events

To identify the prognostic AS events in the GBM SpliceSeq data, the R software base package was used to perform univariate Cox analysis based on the overall survival and PSI values of all eligible samples. The *p*-value calibration in multiple hypotheses testing was performed by the R software “fdrtool” package (false discovery rate (FDR) < 0.05). Kaplan–Meier curves with the log-rank test were performed to compare the overall survival between two subgroups based on the median value of PSI. FDR < 0.05 based on R software “fdrtool” package was considered significant. Venn analysis based on the results of univariate Cox analysis and Kaplan–Meier survival analysis was performed to enhance the reliability of our data. Bubble plots based on the R software “ggplot2” package were used to illustrate the top 20 significant AS events according to the type of AS. An UpSet plot based on the R software “UpSetR” package was used to map the distribution of the 7 different survival-related AS events in all genes.

### Protein-protein interaction analysis

The parent genes of all survivor-related AS events were included in the Retrieval of Interacting Genes/Proteins (STRING) 11.0 database. Correlations (the minimum required interaction score) > 0.9 were included. Disconnected nodes in the network were excluded. The network obtained from the STRING database was then visualized by Cytoscape (version 3.7.1), [28].

### Construction of the AS correlation network

By hand-curated screenings of literature and databases, splicing factors that may play a potentially important role in tumors were identified [29]. The expression levels of splicing factors were derived from transcripts in TCGA. Univariate Cox regression was used to determine the association between the expression levels of splicing factors and the PSI values of survival-related AS events (correlation coefficient > 0.5). The *p*-value calibration in multiple hypotheses testing was performed by the R software “fdrtool” package (FDR < 0.05). All eligible splicing factors and parent genes of corresponding AS events were used to construct the AS correlation network. Weight network diagram was used to visualize the results based on the Cytoscape 3.6.1. Representative dot plots produced with the R software “ggplot2” package were used to visualize the correlation between PSI and splicing factor expression levels for typical AS.

### Construction of the risk prediction model

All samples were randomly divided into training (*n* = 92, accounting for 70% of all samples) and test (*n* = 40, accounting for 30% of all samples) groups by using R software base package. In the training set, survival-related AS events were screened and the AS events whose interquartile spacing values of PSI were less than 0.1 were excluded. Then, the top 20 survival-related AS events with the most significant *P* values were used in LASSO regression to eliminate any potential collinearity. Subsequently, these AS events were included in the multivariate Cox regression analysis and the method of stepwise multiple regression was used for selecting potential prognostic factors (*P* value of inclusion criteria < 0.05, *P* value of exclusion criteria < 0.2). In our model, we first included the AS event with the smallest *P* value which meeting the inclusion criteria (*P* < 0.05), and then we gradually included new variables. Accordingly, after the inclusion of new variables according to the inclusion criteria (*P* < 0.05), we checked whether the *P* value of any variable in the model meet the exclusion criteria (*P* < 0.20), and exclude the corresponding variable if it does not. Final, retained AS events in the multivariate Cox regression analysis were used to construct prognostic models. Coefficients (coef) of AS events in multivariate Cox regression were used as coefficients of corresponding factors in the risk prediction model. The risk value of our model was as follows: risk value = expression of AS event_1_* coef_1_ + expression of AS event_2_* coef_2_ + … + expression of AS event_n_* coef_n_.

The area under the curve (AUC) and the receiver operating characteristic (ROC) based on the testing set were performed to verify the accuracy of the model. All statistical analyses in this study were conducted by using R language (version 3.6.1), and *P* < 0.05 were considered significant.

Kaplan–Meier survival analysis was used to compare the differences of overall survival between the two subgroups based on the median value of PSI; log-rank *P* < 0.05 was considered statistically significant. Univariate and multivariate Cox regression analysis were used to validate whether the obtained risk predictive model was an independent predictor of the outcomes of patients with GBM, and clinical data of patients with GBM were included to calibrate the model.

### Gene set enrichment analysis

We divided samples from TCGA GBM database into low-risk and high-risk subgroups based on medium PSI value. GSEA-4.0.jar was performed to verify whether genes in the two subgroups were rich in an a priori defined set (FDR (qvalue) < 0.25 & *P* < 0.05). The c2.cp.kegg.v7.0.symbols.gmt [Curated] and c2.cp.reactome.v7.0.symbols.gmt [Curated] were selected as annotated gene set.

## Results

### Overview of AS events in GBM

**Fig. 2:**
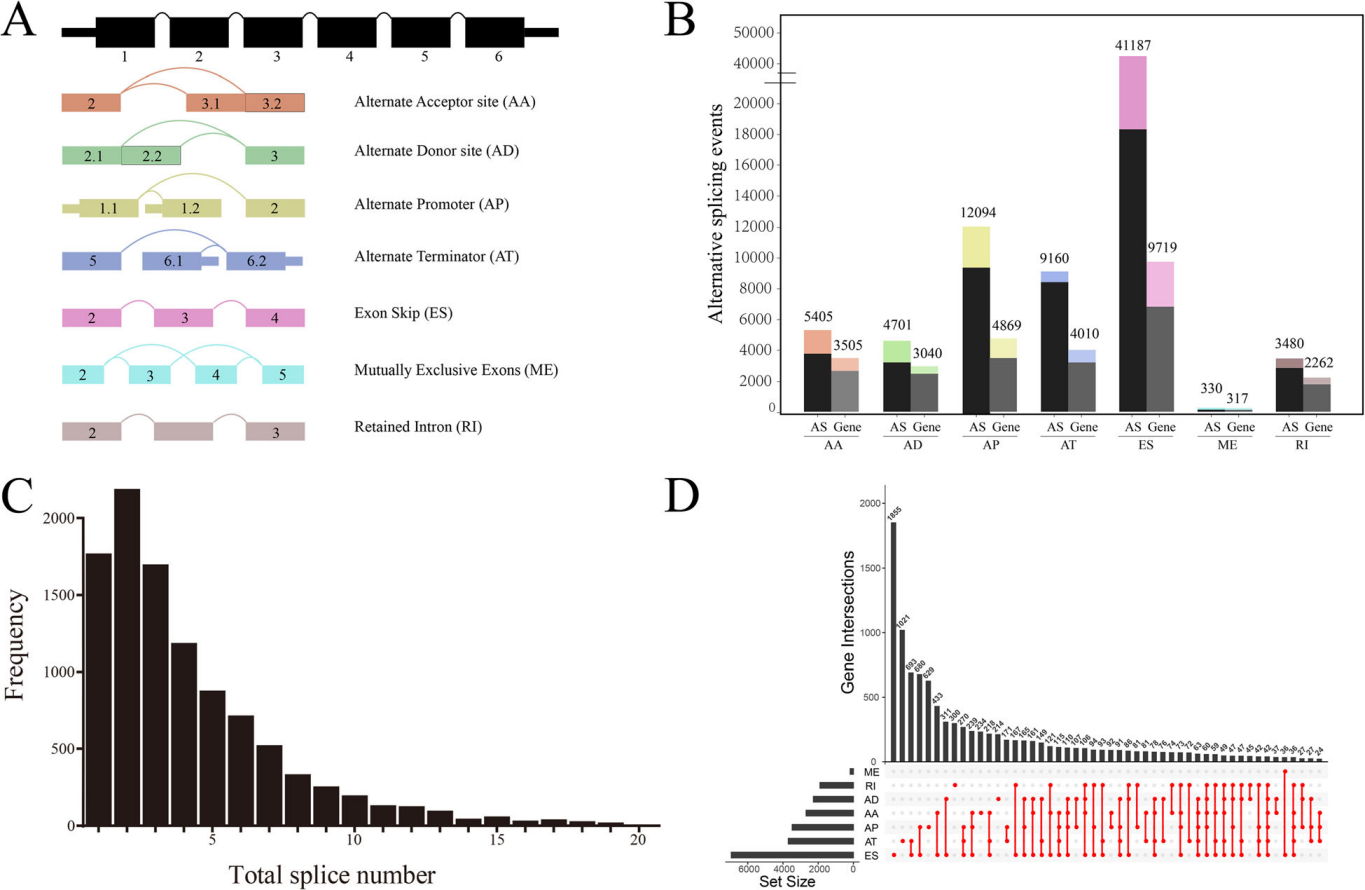
Overview of AS events profiling in GBM. (**A**) Illustrations for seven types of AS events, including Alternate Acceptor site (AA), Alternate Donor site (AD), Alternate Promoter (AP), Alternate Terminator (AT), Exon Skip (ES), Mutually Exclusive Exons (ME), and Retained Intron (RI). (**B**) The number of AS events and involved genes from the GBM patients were depicted according to the AS types. Color bar represents the preliminarily detected AS events and involved genes. Figures above the bar represent the number of preliminarily detected AS events and genes. The Black and gray bar represents the AS events and involved genes filtered by stringent criteria (percentage of samples with PSI value ≥0.75, minimum PSI standard deviation ≥0.01), respectively. (**C**) The frequency distribution of parent genes carrying different AS events. (**D**) UpSet plot of interactions between alternative splicing events and its parent genes

A total of 132 TCGA GBM samples were included in this study, including 86 male and 46 female patients. Their demographic characteristics are shown in Table S1. In our integrated AS events profiling, 76,357 AS events were identified from 12,710 parent genes. Of the seven types of AS events (Fig. 2A), ES occurred most frequently, with 41,187 cases of AS events occurring in 9717 genes (Fig. 2B). Of note is that missing values of PSI were frequent or the variation or dispersion of the PSI value was small in the unfiltered samples. To obtain AS events with potentially physiological effects, a set of strict filters was implemented (percentage of samples with PSI value ≥0.75, minimum PSI standard deviation ≥0.01). 45,610 AS events from 10,433 parent genes were eventually included in our study. We found that a single gene can undergo multiple AS events, with 83.03% of genes undergoing two or more AS events (Fig. 2C). Similarly, 58.86% of genes underwent two or more different types of AS events. An UpSet plot was used to visualize the relationship between parent genes and the occurrence of AS events (Fig. 2D).

### Identification of prognostic AS events

**Fig. 3:**
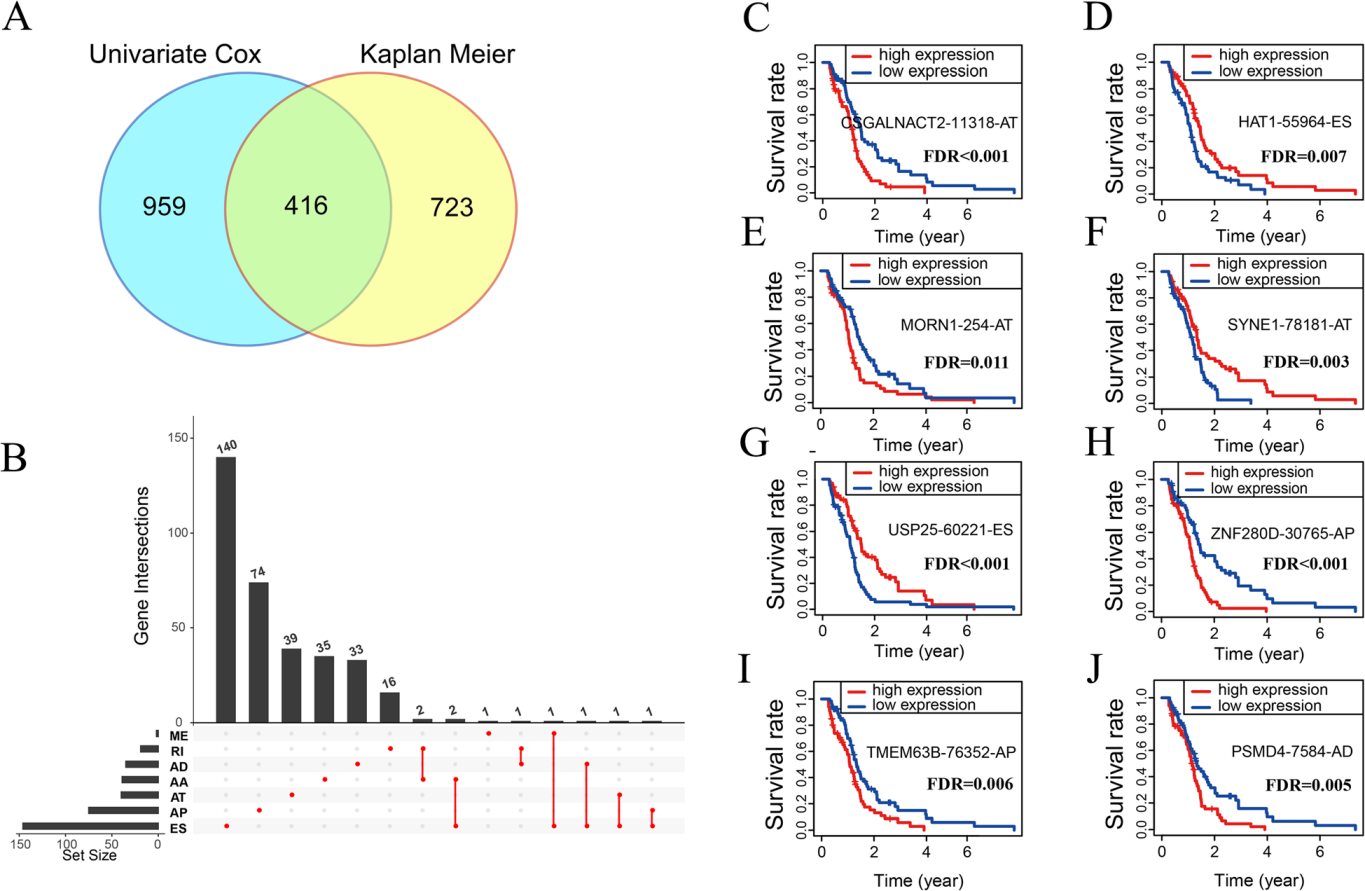
Identification of survival-related AS events in GBM. (**A**) Venn plot of prognosis-related AS events obtained from univariate COX regression and Kaplan-Meier. (**B**) UpSet plot of interactions between survival-related alternative splicing events and its parent genes. (**C-J**) The Kaplan-Meier survival curve of some representative survival-related AS events, including CSGALNACT2|11,318|AT (**C**), HAT1|55,964|ES (**D**), MORN1|254|AT (**E**), SYNE1|78,181|AT (**F**), USP25|60,221|ES (**G**), ZNF280D|30,765|AP (**H**), TMEM63B|76,352|AP (**I**), and PSMD4|7584|AD (**J**)

Survival data have significant clinical value. When the expression level of genes shows statistically significant correlation with prognosis, these genes may be involved in meaningful biological processes of the corresponding disease. Similarly, AS events related to prognosis may also be essential factors in the development and progression of cancer. By intersecting the results of univariate Cox analysis and Kaplan–Meier survival analysis, we obtained a total of 416 survival-related AS events (Fig. 3A; Supplementary Table S2, Table S5). The top 20 AS events for all types or individual type with the most significant *P* values were illustrated in a bubble plot (Fig. S1). Table 1 illustrates concrete details of the top 40 AS events with the most significant *P* values. Similar to the results before screening, the most common type of survival-related AS event was ES, and the least common type was ME. Moreover, among the parent genes of the screened AS events, 93.35% of the genes had only one kind of AS event that was significantly correlated with survival in GBM. Concrete details about the interactions between the seven types of detected AS events are shown in Fig. 3B. The typical Kaplan–Meier curves of survival-related AS events are shown in Fig. 3C–J.

**Table 1:** The detailed information of the top 40 survival-related AS events

AS_id	symbol	splice_type	exons	from_exon	to_exon	novel_splice	pct_with_values	psi_range	std_psi	AveExp	z	HR	HR.95 L	HR.95H	FDR value
78,181	SYNE1	AT	152	null	null	0	1	0.5308	0.0544	0.962424	−4.13214	9.94E-05	1.26E-06	0.00787	3.59E-05
76,352	TMEM63B	AP	1	null	null	0	0.7812	0.6292	0.1019	0.133696	4.04969	77.79737	9.457538	639.9583	5.13E-05
60,221	USP25	ES	20	18	21	0	0.9937	0.225	0.0486	0.072053	−3.82103	0.000223	3.00E-06	0.016666	0.000133
36,699	CDH11	ES	3	2	4	0	1	0.089	0.0169	0.983761	3.816861	1.35E+ 12	797,692.5	2.29E+ 18	0.000135
46,873	HSD11B1L	ES	7	5.3	8	0	0.9875	0.6355	0.1149	0.608853	3.660695	39.97543	5.548568	288.0086	0.000252
36,205	FBXL19	AD	8.2	8.1	9	0	0.9688	0.619	0.0982	0.654726	−3.66063	0.021033	0.00266	0.166284	0.000252
9424	KLHL12	ES	12	11	13	0	1	0.0769	0.0148	0.979878	−3.56496	2.92E-12	1.33E-18	6.41E-06	0.000364
11,318	CSGALNACT2	AT	9	null	null	0	1	0.0611	0.0159	0.986637	3.492296	1.03E+ 11	67,805.11	1.55E+ 17	0.000479
78,886	HDAC9	AP	6	null	null	0	0.8062	1	0.1979	0.186725	3.459482	6.480635	2.248003	18.68264	0.000541
55,964	HAT1	ES	3	2	4	0	1	0.0952	0.0188	0.964809	−3.4369	4.44E-09	7.66E-14	0.000257	0.000588
7584	PSMD4	AD	7.2	7.1	8	0	1	0.0726	0.0137	0.066947	3.328474	8.82E+ 12	210,129.5	3.70E+ 20	0.000873
74,575	CREBRF	AT	10	null	null	0	1	0.0708	0.0157	0.984887	−3.31526	4.56E-13	2.31E-20	9.02E-06	0.000916
76,557	DST	AT	108	null	null	0	1	0.1764	0.0191	0.985881	−3.31112	7.37E-11	7.40E-17	7.33E-05	0.000929
11,956	CCAR1	ES	14	13	15	0	1	0.1978	0.0386	0.960246	3.293613	61,263.4	86.78252	43,248,395	0.000989
16,057	MS4A6A	RI	9.2	9.1	9.3	0	1	0.2982	0.0523	0.339979	−3.27408	0.000744	9.98E-06	0.055515	0.00106
254	MORN1	AT	13	null	null	0	0.975	0.6845	0.1516	0.235858	3.262628	8.502577	2.350391	30.75821	0.001104
30,765	ZNF280D	AP	1	null	null	0	1	0.2587	0.0575	0.869744	3.248248	382.5969	10.57641	13,840.28	0.001161
33,022	CHTF18	AD	10.2	10.1	11	0	0.825	0.352	0.0772	0.091989	−3.24069	0.00656	0.000314	0.13716	0.001192
30,767	ZNF280D	AP	7	null	null	0	1	0.2587	0.0576	0.130101	−3.23772	0.002682	7.44E-05	0.096637	0.001205
68,031	IGF2BP2	ES	11	10	12	0	0.75	0.7263	0.1301	0.637773	3.218231	18.38794	3.121825	108.3073	0.00129
22,747	METTL1	ES	4	3	5	0	1	0.1235	0.0248	0.966295	−3.19472	1.84E-06	5.59E-10	0.006076	0.0014
87,718	CIZ1	ES	6	5	7	0	1	0.1176	0.0222	0.976913	−3.18931	2.03E-06	6.46E-10	0.006397	0.001426
19,257	SLC37A2	ES	12	11	13	0	0.9062	0.2732	0.0556	0.95946	−3.18231	0.002262	5.31E-05	0.096342	0.001461
69,370	HOPX	RI	4.4:4.5	4.3	4.6	0	0.9688	0.6206	0.13	0.645821	3.166331	9.407206	2.349021	37.67336	0.001544
40,976	NKIRAS2	AP	2.1	null	null	0	1	0.2118	0.0306	0.925296	−3.16626	1.86E-07	1.27E-11	0.00273	0.001544
40,977	NKIRAS2	AP	1	null	null	0	1	0.2118	0.0306	0.074703	3.166256	5,366,709	366.3605	7.86E+ 10	0.001544
2784	POMGNT1	RI	22.2	22.1	22.3	0	1	0.08	0.0128	0.983825	−3.15208	3.57E-10	4.77E-16	0.000267	0.001621
69,147	GUF1	ES	9	8	10	1	0.9187	0.431	0.0969	0.742379	−3.13883	0.036717	0.004664	0.289067	0.001696
71,959	PAIP1	AP	2.1	null	null	0	1	0.4933	0.0642	0.947089	−3.12525	0.00011	3.63E-07	0.033446	0.001777
124,660	FYN	ES	11	10	12	1	0.8188	0.7221	0.1231	0.204459	−3.09154	0.027465	0.002812	0.268262	0.001991
11,323	HNRNPF	AP	1	null	null	0	1	0.3749	0.0566	0.109251	−3.0628	0.001469	2.26E-05	0.095475	0.002193
36,979	LRRC29	AP	1	null	null	0	0.975	0.9018	0.1998	0.490236	−3.05767	0.179869	0.059894	0.540167	0.002231
69,344	AASDH	ES	12	11.1	13	0	0.9937	0.4611	0.0827	0.860699	3.023399	47.21881	3.880115	574.6262	0.0025
26,991	NFATC4	RI	12.3	12.2	12.4	0	1	0.7778	0.1157	0.749698	−3.00971	0.063154	0.010452	0.38159	0.002615
88,691	ACOT9	AT	13.2	null	null	0	1	0.1981	0.0369	0.057825	−3.00722	0.000126	3.60E-07	0.0438	0.002636
88,692	ACOT9	AT	19	null	null	0	1	0.1981	0.0369	0.942175	3.007223	7961.978	22.83085	2,776,643	0.002636
64,534	CSPG5	AD	4.2	4.1	5	0	1	0.4724	0.0775	0.134438	−3.0036	0.003635	9.30E-05	0.14202	0.002668
34,421	LYRM1	ES	7	2.2	8.1	0	0.9937	0.4063	0.0723	0.844673	−2.99931	0.008166	0.000353	0.188998	0.002706
100,544	PCBP4	ES	4.1	3	5	1	0.9	0.6469	0.1241	0.469185	2.996804	11.61581	2.33608	57.7579	0.002728
48,957	GPI	AP	2.1	null	null	0	1	0.1481	0.0255	0.955976	2.990991	2,377,718	157.7364	3.58E+ 10	0.002781

### Protein-protein interaction analysis

**Fig. 4:**
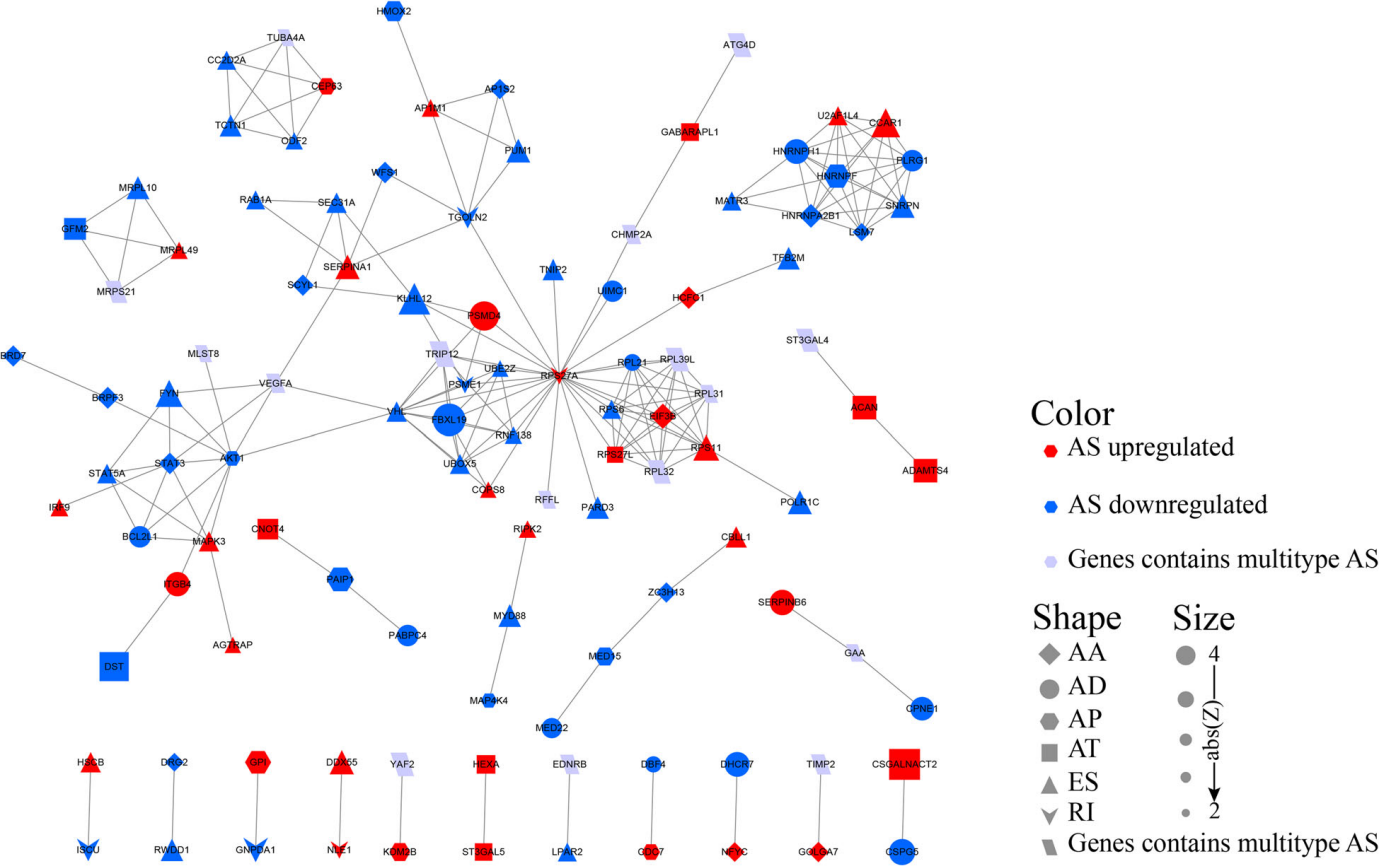
Protein-protein interaction analysis of identified survival-related AS events. Interactome of the 109 parent genes of AS events and 189 edges in the PPI network in GBM. Genes were denoted as nodes in the graph and the interactions between them were presented as edges. The shape, size and color of node respectively represent AS type, the absolute value of Z-score (obtaining from univariate COX regression survival analysis) and change pattern. Exon Skip (ES), Mutually Exclusive Exons (ME), Retained Intron (RI), Alternate Promoter (AP), Alternate Terminator (AT), Alternate Donor site (AD), and Alternate Acceptor site (AA)

AS is thought to have the capability to reconstruct tissue-specific interactions of proteins. It can increase the polymorphism of RNA, which inevitably affects protein function and further modifications. Moreover, abnormal changes of AS in tumors may also uniquely affect protein-protein interactions. The potential mechanism can be elucidated by analyzing the interactions among the corresponding proteins of the AS parent gene. PPI network analysis based on survival AS related genes not only revealed the interaction relationship under normal conditions but also revealed the potential impact of AS events on the whole network (Fig. 4).

### Construction of the AS correlation network and enrichment analysis

**Fig. 5:**
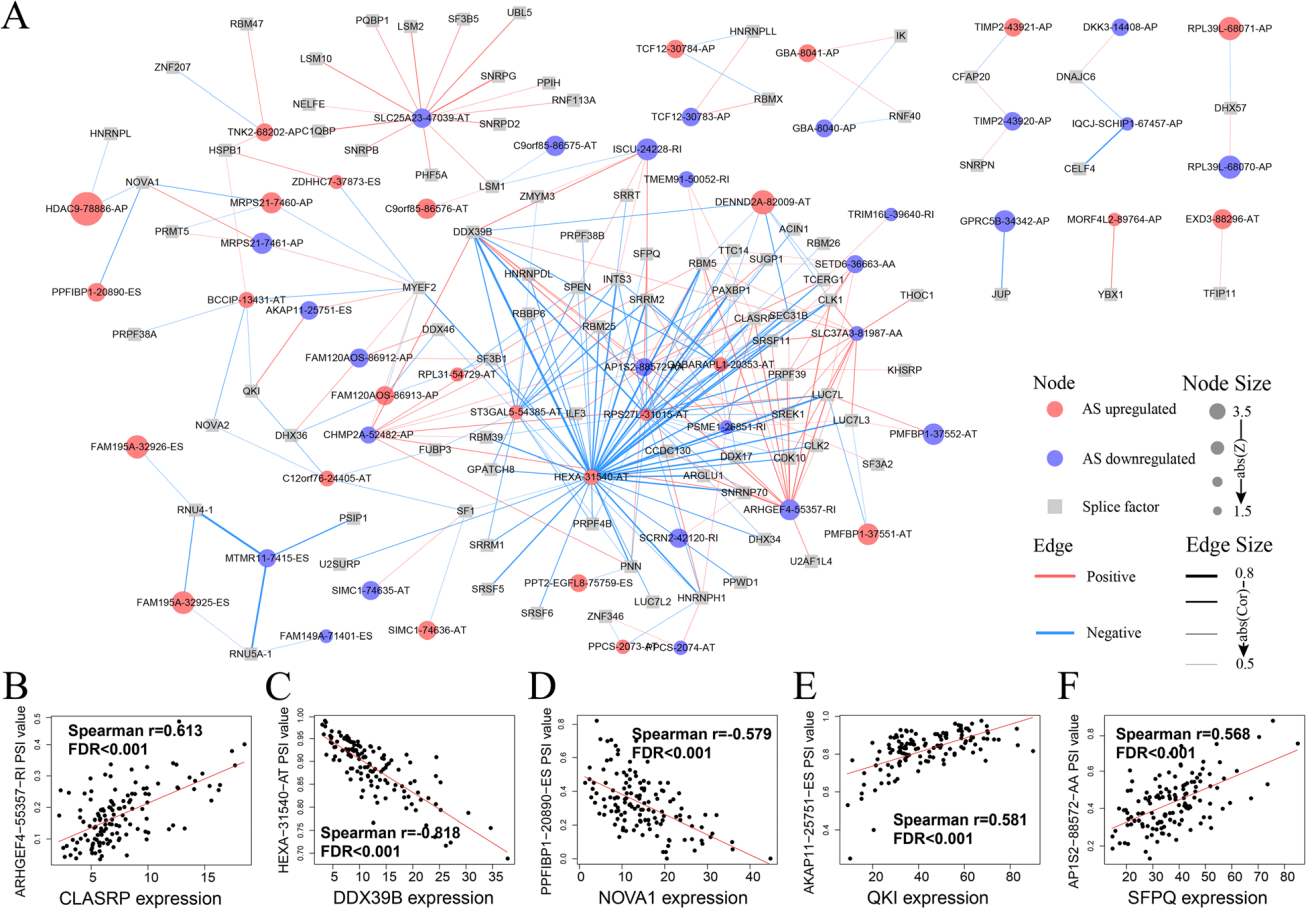
Splicing correlation network in GBM. (**A**) Correlation network between survival related AS events and splicing factors. (**B-F**) Representative dot plots of correlations between expression of splicing factors and PSI values of AS events

The process of AS is regulated by the spliceosome, which is a large and complex molecular machine that removes introns from a transcribed pre-mRNA. Splicing factors are proteins involved in the processing of AS and play a vital role in post-transcriptional regulation. A few key splicing factors may generate large-scale abnormal AS events. Through literature review and database searches, we found 404 splicing factors that had been experimentally verified in studies or predicted by the database to have a potential role in tumors (Table S3). The expression levels of splicing factors were obtained from the TCGA database, and Spearman rank correlation analysis was conducted between all splicing factor expression levels and the PSI value of survival-related AS events (Cor > 0.5, FDR < 0.05). Figure 5A illustrates that the network contains 94 splicing factors, 27 upregulated AS events and 27 downregulated AS events. Among them, a small number of splicing factors, such as DDX39B and SRRM, were correlated with a large number of AS events, which suggested their potential biological functions in GBM. We also noted that typical AS events, such as HEXA-31540-AT, ARHGEF4–55357-RI, and SLC25A23–47,039-AT were associated with 47, 16, and 14 splicing factors, respectively, suggesting that they may be affected by multiple splicing factors to produce different splicing isoforms. The typical correlations between AS events and splicing factors are illustrated in Fig. 5B–F.

Functional enrichment analysis was performed to analyze the potential biological and molecular processes of the genes in the splicing correlation network. Annotated Gene Ontology gene sets such as spliceosomal snRNP assembly (Fisher’s Exact Test, false discovery rate (FDR) < 0.001), regulation of mRNA 3′-end processing (FDR < 0.001), regulation of mRNA metabolic process (FDR < 0.001), and RNA helicase activity (FDR = 0.002) were significantly enriched in the splicing correlation network. In addition, enrichment analysis of Reactome showed the potential correlation between our splicing correlation network and the mRNA Splicing Major Pathway (Fisher’s Exact Test, FDR < 0.001), Cleavage of Growing Transcript in the Termination (FDR < 0.001), and Metabolism of RNA (FDR < 0.001), etc. Consequently, our splicing factors and AS parent genes in the network may play a critical role in multiple biological regulatory activities of GBM (Fig. S2).

### Risk prediction model for GBM patients

To verify the quality of the survival data, we first evaluated the relationship between the clinical characteristics and the survival time of patients. Age at diagnosis (Hazard Ratio (HR) = 1.026, 95% CI: 1.010–1.043, *P* = 0.001), receiving radiotherapy (HR = 0.313, 95% CI: 0.151–0.652, *P* = 0.002), and receiving chemotherapy (HR = 0.326, 95% CI: 0.168–0.634, *P* < 0.001) were significantly associated with OS. Despite the existing censored data, the survival data were still of sufficient clinical value (Table S4).

**Fig. 6:**
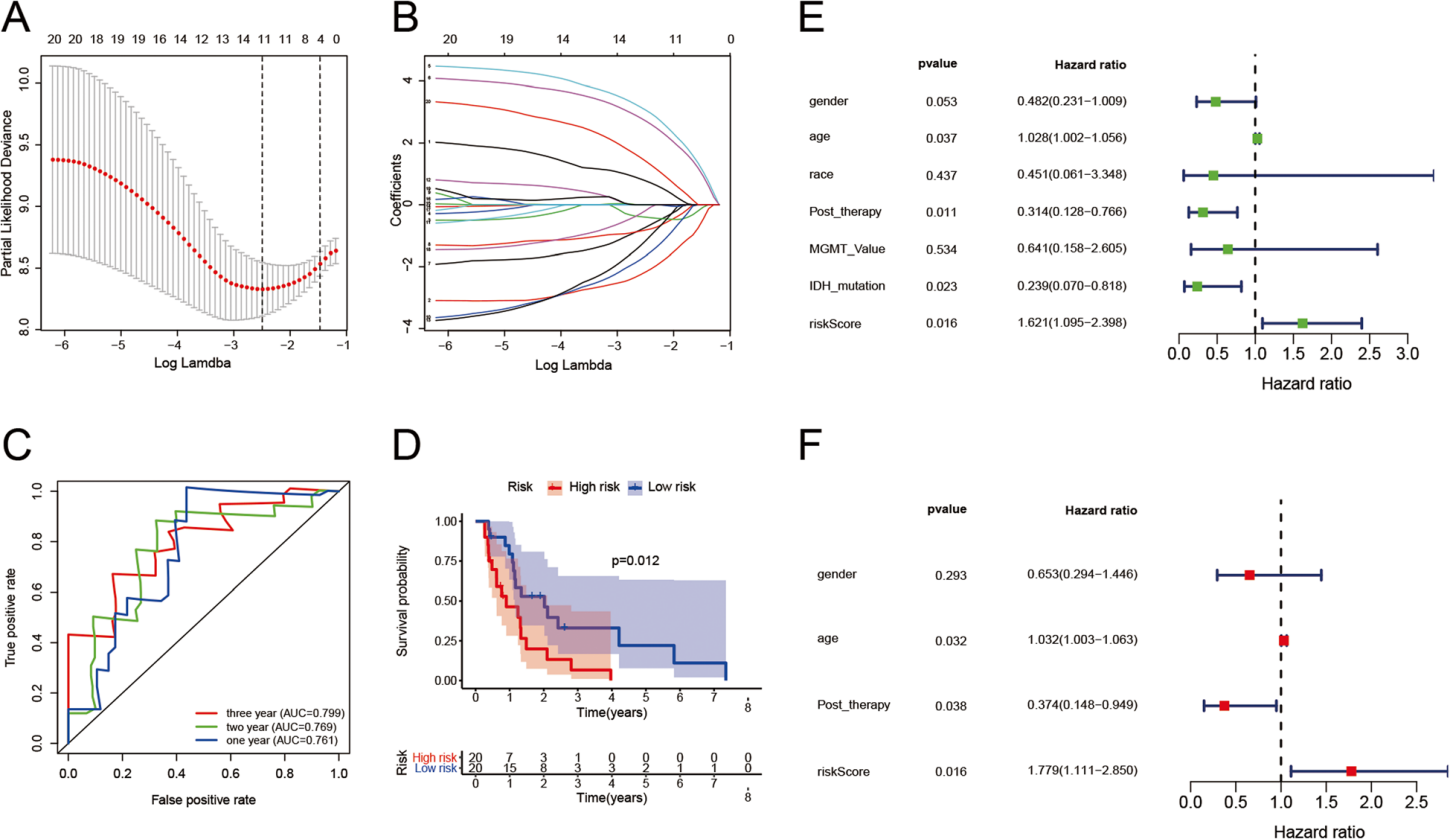
Survival analysis and construction of risk prediction model. (**A-B**) Lasso regression for survival-related AS events based on training set. (**C**) ROC curves of our model in overall survival of one, two and three years based on test set. (**D**) Kaplan-Meier survival curves grouped according to the risk score of our model based on test set. (**E**) univariate Cox regression of survival-related AS events based on test set. (**F**) multivariate Cox regression of remained AS events based on test set

Among the seven types of AS, the top 20 AS events with the most significant *P* values were used as potential prognostic factors. By LASSO regression, we excluded 9 AS events that were significantly collinear with other prognostic factors (Fig. 6A and B). Multivariate Cox regression analysis was used to further screen for independent prognostic factors to construct prognostic models (Supplementary Table S5). Riskscore = β_1_*PSI_AS1_ + β_2_*PSI_AS2_+ … + β_6_*PSI_AS6_ + β_7_*PSI_AS7_ (Supplementary Table S6). The AUC values based on the 1-year, 2-year and 3-year ROC curves were 0.761, 0.769, and 0.799, respectively, indicating moderate performance of the model (Fig. 6C). The TCGA samples were grouped into two groups according to the median value of riskscore, and the results of the Kaplan–Meier survival analysis are shown in Fig. 6D; *P* < 0.05 was considered significant. Potential prognostic factors including gender, age, race, post-therapy, IDH1 mutation status, MGMT status were used to perform univariate Cox analysis, and the results indicated that our risk model could be used as an independent predictor of OS (Fig. 6E). The uneven distribution of IDH1 mutation samples between groups may affect the results of multivariate COX analysis, thus the mutation status of IDH1 was not included in multivariate COX regression analysis (Fig. 6F). Other potential prognostic factors (gender, age, post-therapy) with significant or marginally significant *p* value in the univariate Cox analysis were included in the multivariate Cox analysis. Heatmap of the 7 survival-related AS events of the risk predicted model with prognosis or molecular subtypes is shown in the Fig. S3. To exclude the effect of IDH1 mutation status on the prognosis of patients, Kaplan–Meier survival analysis and multivariate COX regression analysis were performed based on all wild-type IDH1 samples (Fig. S4), which suggest that the predictive efficacy of our risk prediction model was stable in both IDH wild-type population and the total population.

**Fig. 7:**
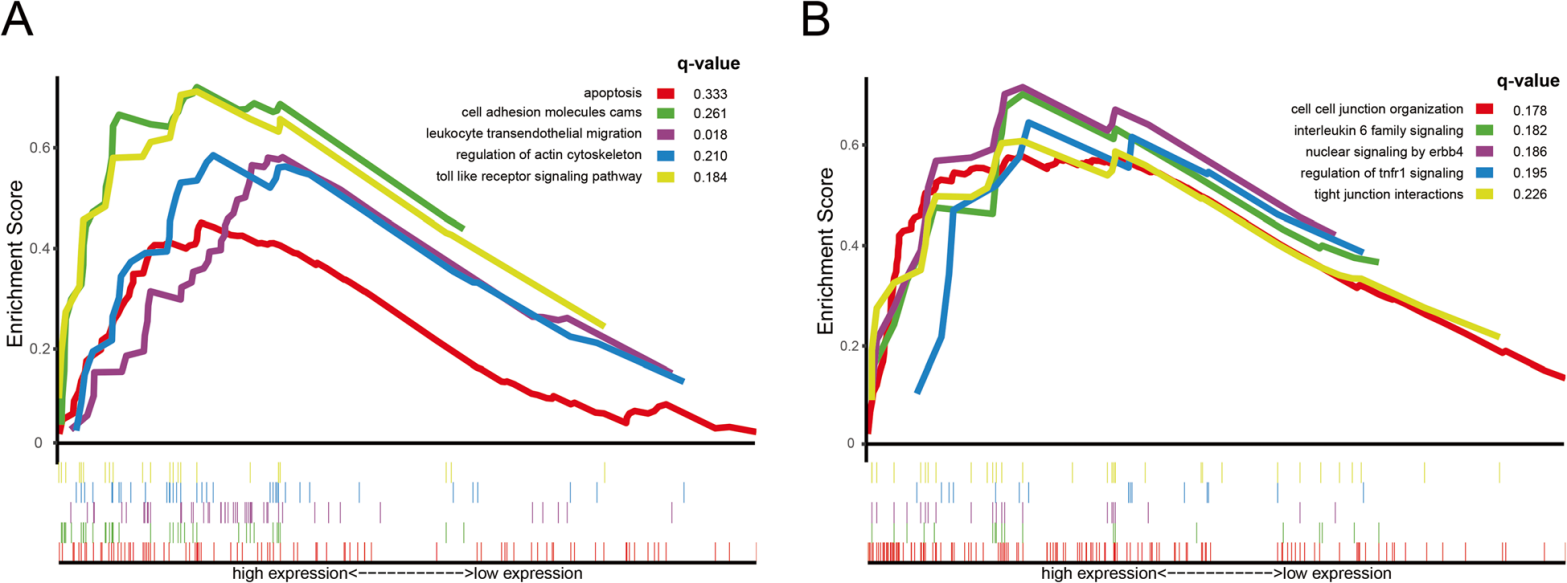
GSEA analysis of the risk prediction model. (**A**) GSEA analysis based on KEGG pathway database. (**B**) GSEA analysis based on Reactome pathway database

GSEA-4.0.jar was performed to verify whether genes in the two subgroups were rich in an a priori defined set (FDR (qvalue) < 0.25 & *P* < 0.05). We select the c2.cp.kegg.v7.0.symbols.gmt [Curated] and c2.cp.reactome.v7.0.symbols.gmt [Curated] as the annotated gene set. And a total of 132 samples were divided into two groups according to the median value of risk value in the prediction model and then GSEA analysis was performed between the two groups. As shown in Fig. 7A and B, the pathways based on the KEGG and Reactome databases were involved in cell adhesion and migration, such as Leukocyte Transendothelial Migration (Enrichment score (ES) = 0.582, NOM *P* < 0.001, FDR q-val = 0.018), Cell Adhesion Molecules (ES = 0.722, NOM *P* = 0.010, FDR q-val = 0.261), Cell-Cell Junction Organization (ES = 0.577, NOM *P* = 0.022, FDR q-val = 0.178), and Tight Junction Interactions (ES = 0.608, NOM *P* = 0.011, FDR q-val = 0.226), and all play a vital role in the biological processes of GBM. In addition, multiple tumor immune-related pathways such as Toll Like Receptor Signaling Pathway (ES = 0.713, NOM *P* = 0.004, FDR q-val = 0.184), Nuclear Signaling by ERBB4 (ES = 0.715, NOM *P* = 0.006, FDR q-val = 0.186) and Interleukin 6 Family Signaling (ES = 0.701, NOM *P* = 0.004, FDR q-val = 0.182) were also active in the GBM process. Detailed information of GSEA results is shown in the Table S7.

## Discussion

AS, as a vital mechanism for the generation of mature mRNA in biological processes, plays an important role in mRNA and protein polymorphism [17]. In malignant diseases, mutations or aberrant expression of splicing factors often leads to abnormal AS. Alsafadi S and colleagues have indicated that SF3B1 is involved in the recognition of corresponding sequences when selecting splice sites in the splicing of RNA and its mutant is the most common mutational component of the spliceosome in cancer [30]. Moreover, abnormal AS plays a significant role in GBM and many other malignant diseases. For example, a dominant negative KAP variant generated by aberrant splicing dysregulated both Cdk2-dependent proliferation and cdc2-dependent migration and increased malignancy in human gliomas [31]. Similarly, MYO1B-fl, an isoform of myosin IB (MYO1B), is regulated by aberrantly expressed SRSF1 and upregulation of MYO1B-fl can strikingly promote cell invasion and proliferation [21]. Moreover, C-CBL, a RING-type ubiquitin E3 ligase, can lead to the downregulation of epidermal growth factor receptor (EGFR) and inhibit cell proliferation in glioma. However, two types of C-CBL isoforms (type I: lacking exon-9 and type II: lacking exon-9 and exon-10) induced by a hypoxic environment contribute to human glioma and its malignant behavior [20]. AS can also act as a tumor suppressor in terms of plasticity in cancer; for example, the USP5 isoform 1 can suppress cell proliferation and invasion, whereas the corresponding USP5 stabilizes the chromatin structure and decreases the synthesis of abnormal proteins [32].

With the rapid development of high-throughput technology, AS, which plays a potentially important role in GBM, has been continuously studied and its relevant pathways and functions have also been explored. Cheung et al. identified 14 genes with differentially variable AS events through genome-wide analysis of exon expression arrays in 24 GBM and 12 nontumor brain samples [33]. More recently, in another large-scale study, Yu and Fu verified 117 genes that differ in PSI values and expression levels in GBM and oligodendroglia and play a role in processes and pathways related to tumor biology [34]. In another study, 2477 genes with alternative exon usage were identified to be associated with GBM, and these genes were simultaneously thought to be involved in multiple GBM related pathways, including cell adhesion, cytoskeleton organization, oxidative phosphorylation, etc. [35]. However, most previous studies have focused on a single gene, and the systematic relationship between AS events and splicing factors in GBM and the relationship between AS and the prognosis of GBM have not been thoroughly discussed.

To the best of our knowledge, the present study is the first systematic identification and analysis of survival-related AS events in GBM tissues. Here, GBM patients’ RNA-seq data, which is more powerful in detecting low expression genes and new splicing variants comparing with microarrays used in previous articles, were used for further analysis. Systematic identification and analysis of survival associated AS events in 76,357 AS and 12,710 genes, which accounts for approximately 66% of human genes, was conducted. Strict inclusion criteria were applied (Percentage of Samples with PSI value ≥75, Average of PSI value ≥0.05), which can make our results more reliable and accurate. Based on our data, 58.86% of parent genes contained more than two types of AS events in the filtered data. However, among the corresponding genes of survival-related AS events, 93.35% only had one type of AS event, which suggested that only a few cases of AS events in GBM are closely related to tumor development and patient prognosis. Therefore, we focused on survival-related AS factors, which may provide valuable clues for seeking potential therapeutic targets as well as prognostic biomarkers.

Our study identified 416 survival-related AS events. Although one single AS event has limited predictive power for GBM, integrated models of multiple AS events can stratify patients’ prognosis with great accuracy. Of note is that GSEA showed that AS events in our model were mainly active in the pathways related to proliferation, migration, apoptosis, and tumor immunity, which may indicate that abnormal AS mainly affected tumor biological processes through these pathways.

Although we focused on the AS events in the above risk model, all survivor-related AS events have potential prognostic value. Therefore, a regulatory network composed of splicing factors’ expression levels and PSI values of AS events can provide a more systematic understanding of AS and related pathways in GBM. In our splicing correlation network, multiple splicing factors, such as DDX39B and SRRM, and multiple AS events, such as HEXA-31540-AT and ARHGEF4–55357-RI, were widely connected in the network. This indicated that these AS events and splicing factors interact actively in the network and may play important roles in the malignant behavior of tumors. For example, DDX39B is a potential therapeutic target in prostate cancer, and its expression imbalance may lead to multiple tumorigenesis events [36].

Considering the high incidence of abnormal AS events in cancer, small molecule drugs targeting specific AS events or splicing factors represent a potential promising new therapeutic strategy in cancer therapy. A recent article described the role of many small molecule modulators targeting specific AS events or splicing factors in cancer therapy, including FR901464, E7107, AR-A014418, etc. [37] Therefore, our study can provide some potential targets for the treatment of GBM.

In our research, we conducted a genome-wide RNA AS profiling based on a large sample of GBM tissues. Additionally, novel AS biomarkers and clinically useful prediction model were presented in our study. However, some limitations still need to be noted. First, the lack of control data from para-carcinoma tissue in this study may negatively affect the sensitivity and specificity of the results. Second, due to the extensive heterogeneity of GBM in space, there may be variation in the PSI values of AS events in different parts of the same GBM sample. Data from a small sample cannot represent the full landscape of GBM. However, most TCGA GBM PSI data are derived from sequencing data of single sampling and the heterogeneity of GBM may be an uncontrollable confounder, which leads to a decrease in the reliability of our prognostic model. Further functional and clinical trials are needed to determine the pathway between the splicing factors and AS events and the clinical utility of the risk prediction model.

## Conclusion

In summary, our study systematically identified survival associated AS events and expounded on the potential regulatory relationships between survivor-related AS events and splicing factors. Our study is a foundation for further exploring GBM-related AS therapeutic targets and prognostic factors, and the AS-related risk prediction model we constructed also provides predictive value for the clinical outcomes of patients with GBM.

## Supplementary Information

**Additional file 1 Table S1.** Clinical features for the GBM patients in the TCGA cohort.

**Additional file 2 Table S2**. The detailed information of the survival-related AS events.

**Additional file 3 Table S3**. The detail of the 404 splicing facters which had been experimentally validated or predicted by databases.

**Additional file 4 Table S4**. Clinical Prognostic predictor for GBM patients.

**Additional file 5 Table S5**. Flow chart for screening significant AS events of the risk prediction model.

**Additional file 6 Table S6**. Details of the formula used to calculate the risk score.

**Additional file 7 Table S7.** Detailed information of GSEA results.

**Additional file 8 Fig. S1** Bubble plot of top 20 survival associated AS events for different types. (**A-G**) bubble plots of top 20 survival associated AS events for AA(**A**), AD(**B**), AP(**C**), AT(**D**), ES(**E**), ME(**F**), and RI(**G**). **Fig. S2**. Pathway analysis and the regulation network between splicing factors and survival-related AS events of genes involved. (**A**) Gene ontology analysis for biological processes, cellular components, and molecular functions. (**B**) Reactome pathway analysis between splicing factors and survival-related AS events of genes involved. **Fig. S3** Heatmap of the 7 survival-related AS events of the risk predicted model with prognosis and molecular subtypes. All 132 samples were included in the analysis. Each cluster has corresponding annotations. For the value of post-therapy, 0 means receiving no postoperative therapy, 1 means receiving only postoperative radiotherapy or receiving only postoperative chemotherapy, 2 means receiving both postoperative radiotherapy and chemotherapy. **Fig. S4** Kaplan-Meier survival analysis and ROC curves of multivariate COX analysis for 34 wild-type IDH1 samples. (**A**) Kaplan-Meier survival curves for wild-type IDH1 samples grouped according to the risk score of our model. (**B**) ROC curves of wild-type IDH1 samples in overall survival of one, two and three years.

## Data Availability

All data obtained and used during this study can be found in the TCGA (http://www.cbioportal.org) and TCGA SpliceSeq databases (https://bioinformatics.mdanderson.org/TCGASpliceSeq/). Public access to all databases used in this study is open.

## Abbreviations

AS: Alternative splicing
GBM: Glioblastoma
mRNA: Message RNAs
TCGA: The Cancer Genome Atlas
CNV: Copy number variation
lncRNA: Long non-coding RNA
PSI: Percent Spliced In
AA: Alternate acceptor site
AD: Alternate donor
AP: Alternate promoter
AT: Alternate terminator
ES: Exon skip
ME: Mutually exclusive exon
RI: Retained intron
Coef: Coefficients
AUC: Area under the curve
ROC: Receiver operating characteristic curve
GSEA: Gene set enrichment analysis
KEGG: Kyoto Encyclopedia of Genes and Genomes
OS: Overall survival
FDR: False discovery rate
HR: Hazard Ratio
ES: Enrichment score
EGFR: Epidermal growth factor receptor
