# Supplementary material for: Genome-wide profiling of alternative splicing in glioblastoma and their clinical value
Source: BMC Cancer. 2021 Aug 26;21:958. doi: 10.1186/s12885-021-08681-z (PMC8393481; doi:10.1186/s12885-021-08681-z)
Supplement: Supplementary file 1 — Additional file 1 Table S1. Clinical features for the GBM patients in the TCGA cohort. [file 12885_2021_8681_MOESM1_ESM.docx]

**Table S1: Clinical features for the GBM patients in the TCGA cohort.**

| Characteristics | | | No. of patients (%) | |
| --- | --- | --- | --- | --- |
|  |  |  | (n = 132) | |
| Age (years) | | |  |  |
|  | Median(IQR) | | 59.3(21-85) | |
|  | < 60 | | 62(46.97%) | |
|  | ≥ 60 | | 70(53.03%) | |
| Sex | | |  |  |
|  | Female | | 46(34.85%) | |
|  | Male | | 86(65.15%) | |
| Race | | |  |  |
|  | White | | 122(92.42%) | |
|  | Black_or_African_American | | 5(3.79%) | |
|  | Asian |  | 4(3.03%) | |
|  | NA |  | 1(0.76%) | |
| Chemotherapy | | |  |  |
|  | Yes | | 113(85.61%) | |
|  | No | | 11(8.33%) | |
|  | NA |  | 8(6.06%) | |
| Radiotherapy | | |  |  |
|  | Yes | | 118(89.39%) | |
|  | No | | 8(6.06%) | |
|  | NA |  | 6(4.55%) | |
| Survival status | | |  |  |
|  | No | | 104(78.79%) | |
|  | Yes | | 28 (21.21%) | |
