# Supplementary material for: Genome-wide profiling of alternative splicing in glioblastoma and their clinical value
Source: BMC Cancer. 2021 Aug 26;21:958. doi: 10.1186/s12885-021-08681-z (PMC8393481; doi:10.1186/s12885-021-08681-z)
Supplement: Supplementary file 4 — Additional file 4 Table S4. Clinical Prognostic predictor for GBM patients. [file 12885_2021_8681_MOESM4_ESM.docx]

**Supplementary Table S4. Clinical Prognostic predictor for GBM patients.**

| id | HR | HR.95L | HR.95H | P value |
| --- | --- | --- | --- | --- |
| gender | 0.857447 | 0.570251 | 1.289284 | 0.459895 |
| age | 1.026295 | 1.010219 | 1.042627 | 0.001273 |
| race | 0.607573 | 0.222741 | 1.657284 | 0.330431 |
| Post_therapy | 0.539288 | 0.376589 | 0.772279 | 0.000751 |
