# Supplementary material for: Genome-wide profiling of alternative splicing in glioblastoma and their clinical value
Source: BMC Cancer. 2021 Aug 26;21:958. doi: 10.1186/s12885-021-08681-z (PMC8393481; doi:10.1186/s12885-021-08681-z)
Supplement: Supplementary file 7 — Additional file 7 Table S7. Detailed information of GSEA results. [file 12885_2021_8681_MOESM7_ESM.docx]

| **Table S7: Detailed information of GSEA results** | | | |  |  |
| --- | --- | --- | --- | --- | --- |
| **KEGG (c2.cp.kegg.v7.0·symbols.gmt)** | | |  |  |  |
| name | SIZE | ES | NES | NOM p-val | FDR q-val |
| leukocyte transendothelial migration | 116 | 0.582 | 2.138 | 0.000 | 0.018 |
| toll like receptor signaling pathway | 102 | 0.713 | 1.833 | 0.004 | 0.184 |
| regulation of actin cytoskeleton | 213 | 0.587 | 1.847 | 0.008 | 0.210 |
| cell adhesion molecules cams | 131 | 0.722 | 1.771 | 0.010 | 0.261 |
| apoptosis | 87 | 0.453 | 1.648 | 0.033 | 0.333 |
| **Reactome (c2.cp.reactome.v7.0·symbols.gmt)** | | | |  |  |
| name | SIZE | ES | NES | NOM p-val | FDR q-val |
| cell cell junction organization | 65 | 0.577 | 1.835 | 0.022 | 0.178 |
| interleukin 6 family signaling | 24 | 0.701 | 1.837 | 0.004 | 0.182 |
| nuclear signaling by erbb4 | 32 | 0.715 | 1.819 | 0.006 | 0.186 |
| regulation of tnfr1 signaling | 33 | 0.645 | 1.844 | 0.008 | 0.195 |
| tight junction interactions | 30 | 0.608 | 1.855 | 0.011 | 0.226 |
