# Supplementary material for: Genome-wide profiling of alternative splicing in glioblastoma and their clinical value
Source: BMC Cancer. 2021 Aug 26;21:958. doi: 10.1186/s12885-021-08681-z (PMC8393481; doi:10.1186/s12885-021-08681-z)
Supplement: Supplementary file 8 — Additional file 8 Fig. S1 Bubble plot of top 20 survival associated AS events for different types. (A-G) bubble plots of top 20 survival associated AS events for AA(A), AD(B), AP(C), AT(D), ES(E), ME(F), and RI(G). Fig. S2. Pathway analysis and the regulation network between splicing factors and survival-related AS events of genes involved. (A) Gene ontology analysis for biological processes, cellular components, and molecular functions. (B) Reactome pathway analysis between splicing factors and survival-related AS events of genes involved. Fig. S3 Heatmap of the 7 survival-related AS events of the risk predicted model with prognosis and molecular subtypes. All 132 samples were included in the analysis. Each cluster has corresponding annotations. For the value of post-therapy, 0 means receiving no postoperative therapy, 1 means receiving only postoperative radiotherapy or receiving only postoperative chemotherapy, 2 means receiving both postoperative radiotherapy and chemotherapy. Fig. S4 Kaplan-Meier survival analysis and ROC curves of multivariate COX analysis for 34 wild-type IDH1 samples. (A) Kaplan-Meier survival curves for wild-type IDH1 samples grouped according to the risk score of our model. (B) ROC curves of wild-type IDH1 samples in overall survival of one, two and three years. [file 12885_2021_8681_MOESM8_ESM.zip › Figure legend for supplementary figuresR4.docx]

Fig. S1. Bubble plot of top 20 survival associated AS events for different types. (a-g) bubble plots of top 20 survival associated AS events for AA(a), AD(b), AP(c), AT(d), ES(e), ME(f), and RI(g).

Fig. S2. Pathway analysis and the regulation network between splicing factors and survival-related AS events of genes involved. (a) Gene ontology analysis for biological processes, cellular components, and molecular functions. (b) Reactome pathway analysis between splicing factors and survival-related AS events of genes involved.

Fig. S3. Heatmap of the 7 survival-related AS events of the risk predicted model with prognosis and molecular subtypes. All 132 samples were included in the analysis. Each cluster has corresponding annotations. For the value of post-therapy, 0 means receiving no postoperative therapy, 1 means receiving only postoperative radiotherapy or receiving only postoperative chemotherapy, 2 means receiving both postoperative radiotherapy and chemotherapy.

Fig. S4. Kaplan-Meier survival analysis and ROC curves of multivariate COX analysis for 34 wild-type IDH1 samples. (a) Kaplan-Meier survival curves for wild-type IDH1 samples grouped according to the risk score of our model. (b) ROC curves of wild-type IDH1 samples in overall survival of one, two and three years.
